# B cells in systemic lupus erythematosus: Targets of new therapies and surveillance tools

**DOI:** 10.3389/fmed.2022.952304

**Published:** 2022-08-30

**Authors:** Ioannis Parodis, Mariele Gatto, Christopher Sjöwall

**Affiliations:** ^1^Division of Rheumatology, Department of Medicine Solna, Karolinska Institutet and Karolinska University Hospital, Stockholm, Sweden; ^2^Department of Rheumatology, Faculty of Medicine and Health, Örebro University, Örebro, Sweden; ^3^Unit of Rheumatology, Department of Medicine, University of Padua, Padua, Italy; ^4^Division of Inflammation and Infection, Department of Biomedical and Clinical Sciences, Linköping University, Linköping, Sweden

**Keywords:** systemic lupus erythematosus, B cells, B lymphocyte, plasma cells, plasmablasts, therapy, biologics, lupus nephritis

## Abstract

B cell hyperactivity is a hallmark of the complex autoimmune disease systemic lupus erythematosus (SLE), which has justified drug development focusing on B cell altering agents during the last decades, as well as the off-label use of B cell targeting biologics. About a decade ago, the anti-B cell activating factor (BAFF) belimumab was the first biological agent to be licensed for the treatment of adult patients with active yet non-renal and non-neuropsychiatric SLE, to later be expanded to include treatment of pediatric SLE and, recently, lupus nephritis. B cell depletion is recommended as an off-label option in refractory cases, with the anti-CD20 rituximab having been the most used B cell depleting agent to date while agents with a slightly different binding specificity to CD20 such as obinutuzumab have also shown promise, forming a part of the current pipeline. In addition, terminally differentiated B cells have also been the targets of experimental therapies, with the proteasome inhibitor bortezomib being one example. Apart from being promising drug targets, B and plasma cells have also shown promise in the surveillance of patients with SLE, especially for monitoring B cell depleting or B cell altering therapies. Inadequate B cell depletion may signify poor expected clinical response to rituximab, for example, while prominent reductions in certain B cell subsets may signify a protection against flare development in patients treated with belimumab. Toward an era with a richer therapeutic armamentarium in SLE, including to a large extent B cell altering treatments, the challenge that emerges is to determine diagnostic means for evidence-based therapeutic decision-making, that uses clinical information, serological markers, and gene expression patterns to guide individualized precision strategies.

## Introduction

Systemic lupus erythematosus (SLE) is a chronic autoimmune inflammatory disease that is characterized by a multiple organ involvement and a prominent heterogeneity of clinical symptoms and disease severity (1). While the treatment of SLE has been non-targeted for many years, mainly comprising antimalarial agents (2), glucocorticoids, and broad immunosuppressants that hamper inflammation in a non-specific manner (3), more selective therapeutic modalities have been used for SLE during the last decades (4), including B cell targeting therapies (5).

Belimumab, a monoclonal antibody that binds to the soluble form of the B cell activating factor belonging to the tumor necrosis family (BAFF; also known as B lymphocyte stimulator, BLyS), was approved for the treatment of SLE in 2011 after two successful phase III clinical trials (6, 7); it was the first in history biological agent to be approved for SLE (8), lending credence to the notion that B cells are central in the pathogenesis of SLE (9). Rituximab, an anti-CD20 B cell depleting monoclonal antibody has been used as an off-label therapeutic option since even earlier (10, 11), and is still used in difficult to treat or refractory cases despite serial failures of clinical trials to show added value on top of standard therapy (3). In this review, we summarize evidence for B cell and plasma cell targeting therapies in SLE, and advocate for the use of B cells as important tools not only as treatment targets, but also disease surveillance.

Loss of self-tolerance is a distinctive feature in SLE pathogenesis, leading up to immune responses toward endogenous nuclear and cytoplasmic components. Terminally differentiated antibody-secreting B cells i.e., plasmablast and long-lived plasma cell clones constitute the main producers of autoantibodies against such endogenous antigens, contributing to tissue injury and maintenance of the inflammatory milieu through immune complex formation and induction of type I interferon-mediated proinflammatory cytokine production. Autoantibodies such as anti-double stranded DNA (anti-dsDNA) and anti-Smith (Sm) antibodies are considered fairly specific for SLE and are associated with clinical disease features (12), while circulating anti-dsDNA levels oftentimes follow SLE disease activity (13, 14). Importantly, autoantibody production is not the only mechanism through which B cells contribute to SLE pathogenesis, as evidenced in murine lupus models where B cells that did not secrete autoantibodies still were important for disease progression (15); thus, the constantly hyperactive B cell lineages in SLE are also important for antigen presentation to T cells and cytokine secretion (16), or expression of toll-like receptors (TLRs) (17).

To characterize B cells, surface markers are used, such as CD19, CD20, and CD22, expressed at different stages of B cell maturation. B cell responses in SLE are currently hampered by two main means i.e., (i) BAFF inhibition through belimumab, and (ii) B cell depletion through anti-CD20 agents, such as rituximab, obinutuzumab, and ofatumumab. BAFF is a member of the tumor necrosis factor ligand superfamily and is mainly produced by myeloid and stromal cells (18). BAFF contributes to B cell survival, proliferation, and antibody production, through binding to three receptors expressed on the surface of B cells at different maturation stages i.e., the BAFF-Receptor (BAFF-R; also known as BLyS receptor 3, BR3), transmembrane activator and calcium modulator and cyclophilin ligand interactor (TACI), and B cell maturation antigen (BCMA) (19). Notably, BAFF levels have been demonstrated to be elevated in patients with SLE compared with healthy controls and show correlates with disease activity (20–23). Apart from BAFF, B cell stimulation is also induced by cytokines such as type I interferons, interleukin (IL)-6, IL-21, as well as a proliferation-inducing ligand (APRIL), the latter mainly contributing to plasma cell survival (24, 25).

## Targeting B cells to treat SLE

Early uncontrolled studies of the chimeric anti-CD20 monoclonal antibody rituximab in SLE showed promise, but two phase III randomized controlled trials (RCTs), the EXPLORER trial in non-renal SLE (26) and the LUNAR trial in lupus nephritis (27), failed to meet their primary endpoints (28). This notwithstanding, rituximab has been used off-label for the treatment of cases of SLE and lupus nephritis that have not been responsive to standard therapy (3, 29, 30). While lupus nephritis, a potentially life-threatening condition (31), has been the most common SLE manifestation managed with rituximab in several centers, rituximab has also been used to treat severe polyarthritis, hematological SLE disease, especially severe haemolytic anemia or autoimmune thrombocytopenia, and neuropsychiatric SLE (32–37). However, it is worth noting that rituximab use has also raised concerns regarding adverse events, such as infusion-related reactions (38–40), late-onset neutropenia (41), and hypogammaglobulinaemia (42, 43).

In addition to rituximab, the humanized anti-CD20 monoclonal antibody ocrelizumab was evaluated in a phase III trial of severe lupus nephritis, which however was terminated due to serious infections (44); for this reason, ocrelizumab was not studied further in the context of SLE. The humanized anti-CD20 monoclonal antibody obinutuzumab has a different binding specificity to the CD20 molecule, resulting in superior cytotoxic effects for B cells over rituximab (45); obinutuzumab was studied in a phase II trial of lupus nephritis and showed ability to induce complete renal response (46). The prospect of obinutuzumab for the treatment of lupus nephritis is currently tested in a phase III clinical trial (NCT04221477). Ofatumumab is a fully human anti-CD20 monoclonal antibody that is approved for the treatment of chronic lymphocytic leukemia (47) and has shown promise in small studies of SLE, mainly used to treat lupus nephritis, immune-mediated thrombocytopenia, and autoimmune haemolytic anemia (48–51). Obinutuzumab and ofatumumab are considered viable options for patients in whom rituximab was effective but adverse events, namely infusion reactions, resulted in discontinuation (49), or for patients in whom rituximab did not induce complete B cell depletion (39).

Lastly, the humanized monoclonal antibody epratuzumab is directed against CD22, and while it showed promising results regarding both tolerability and efficacy in a phase IIb trial (52, 53), two subsequent phase III trials failed to prove efficacy (54).

## Targeting plasma cells

While most B cell targeting drugs currently used in SLE mainly exert their effects on B cells of early maturation stages, plasmablasts, and short-lived plasma cells (55), proteasome inhibition affects long-lived plasma cells and has been used in severe therapy-resistant SLE cases (56–60). Proteasome inhibition results in accumulation of defective immunoglobulin chains, endoplasmic reticulum stress, misfolded proteins, and ultimately plasma cell apoptosis (61, 62). Being large-scale antibody producers, long-lived plasma cells are highly sensitive to proteasome inhibition (58). In addition, proteasome inhibitors also hamper the production of pro-inflammatory cytokines through regulation of NF-κB signaling (63).

Bortezomib, a dipeptide containing a boron atom that exerts its effects on plasma cells by binding with high affinity and specificity the catalytic site of the 26S proteasome, is approved for the treatment of mantle cell lymphoma and multiple myeloma (64). The prospect of bortezomib in SLE (65) gained promise after experiments in murine lupus, and reports of bortezomib use to treat allograft rejection after kidney transplantation (66, 67). In a recent nationwide study from Sweden, the current clinical experience based on 12 patients who received bortezomib for refractory SLE was reported (68). The therapeutic effects of bortezomib combined with glucocorticoids were beneficial in a majority of the patients with severe and life-threatening SLE manifestations that had been irresponsive to conventional immunosuppressive agents; these effects included alleviation of proteinuria levels and complement consumption, as well as seroconversion from positive to negative anti-dsDNA antibody status (68). Safety signals that emerged in this study mainly comprised the potential risk for infections and hypogammaglobulinemia (68).

On the other hand, concerns may argue that sole bortezomib treatment might not be as sufficient in autoimmune conditions as in non-autoimmune hematological diseases, due to a rapid bone marrow repopulation with short-lived plasma cells and subsequent rise in autoantibody levels that occur upon bortezomib withdrawal, which is likely attributable to the limited effects of bortezomib on bone marrow B and T cell precursors (62). Additionally, the only randomized trial that employed bortezomib in SLE did not demonstrate superiority of bortezomib over placebo in clinical or serological efficacy (69). Nevertheless, careful interpretation of these results should be entailed due to the limited number of patients enrolled and the choice of endpoints. In fact, proteasome inhibition in SLE might be an option in severe, refractory cases (68), and might require additional subsequent B cell depleting modalities to ensure long-term efficacy. In this regard, plasma cells may also by targeted through blockade of CD38. Daratumumab is a monoclonal antibody against CD38 approved for multiple myeloma, which has been successfully used in patients with refractory SLE who previously had received bortezomib (70), lending merit for targeting terminally differentiated B cells in recalcitrant forms of SLE using one or multiple agents.

## Targeting B cell-related cytokines

BAFF has been a central target molecule in drug development for SLE, due to is key roles in B cell homeostasis. Targeting BAFF has been proven a successful therapeutic strategy with the approval of the anti-BAFF monoclonal antibody belimumab, formerly also known as Lympho-Stat B, after two successful phase III RCTs (6, 7). In different studies, patients with low B cell counts, high BAFF levels, serologically active disease, limited or no established organ damage, and no exposure to cigarette smoking have been shown to be more benefited from belimumab therapy (71–80). Safety signals reported during belimumab therapy mostly concern infusion reactions and mild infectious events (6, 7, 72, 81), and some caution was raised during the early post-market phase about the risk of psychiatric disorders (82), but the drug has shown an excellent overall safety profile, also over a longer term (83).

Another BAFF-inhibiting agent is atacicept, a receptor construct that combines TACI with the Fc fraction of human IgG, thus inhibiting both BAFF and APRIL (84). Atacicept was evaluated in a clinical trial of lupus nephritis, which was terminated prematurely due to adverse events such as hypogammaglobulinemia and infections (85); however, the development of atacicept for the treatment of SLE has not been fully abandoned.

The fusion protein blisibimod consists of four high-affinity BAFF-binding domains and the Fc domain of human IgG1; in contrast to belimumab that only binds to the soluble counterpart of BAFF, blisibimod targets both soluble and membrane-bound BAFF. Following a promising dose-ranging phase IIb clinical trial of SLE that determined a safe and effective dose (86), a subsequent phase III clinical trial failed unfortunately to prove efficacy (87).

Tabalumab is a fully human monoclonal antibody that also targets both soluble and membrane-bound BAFF. Two phase III clinical trials of tabalumab in SLE have been completed, of which only one met its primary endpoint (88, 89), being the reason why its development for SLE was stopped. However, it is worth noting that key secondary endpoints were met in both trials, justifying the rationale of targeting both the cleaved and membrane-bound BAFF molecule (90, 91).

Anifrolumab is a recently developed monoclonal antibody against the type I interferon (IFN) receptor (IFNAR), hence with inhibitory capability on the type I IFN pathway. While type I IFNs are mainly produced by plasmacytoid dendritic cells (pDCs) and resident cells in target tissues (92), B cells are highly sensitive to the IFN pleiotropic action and are prompted to differentiation and activation upon IFN-mediated signaling (24). The successful phase IIb trial of anifrolumab in patients with moderate or severe SLE (93) put the grounds for the conception of patient stratification based on endotypic features, e.g., the magnitude of the IFN signature, and in contrast to the failure of the first phase III trial of anifrolumab in SLE (TULIP1) (94), the drug was successful in the subsequent phase III trial TULIP2 (95). Based on an overall evaluation of the primary and key secondary endpoints of the TULIP trials, anifrolumab received approval by regulatory authorities for use in SLE. Additionally, a phase II trial of anifrolumab in active lupus nephritis showed initial promise (96) and prompted an ongoing phase III trial. Safety signals reported for anifrolumab therapy mostly concern reactivation of opportunistic infections, particularly by varicella zoster virus (VZV), due to the IFN blockade, especially in patients with kidney involvement (97).

While the therapeutic armamentarium for SLE has been enriched with new therapeutic modalities that directly or indirectly target B cells of diverse maturation stages, the identification of the best patient candidates for the different treatments remains lacunose. Clinical features and traditional serological markers (71–80), as well as particular immunological signatures (94, 95), have been introduced as potential predictors of therapeutic responses, representing important steps toward more informed decision-making processes, yet optimisation is still limited by major knowledge gaps regarding in-depth characterization of underlying mechanisms, and the impact those mechanisms have on the individual patient.

## Combination therapies to target B cells

The observed rise in BAFF levels upon treatment with rituximab (98) has been speculated to be one of the reasons why clinical trials of rituximab in SLE have failed, providing grounds to the rationale for combining rituximab with belimumab to achieve better B cell responses (23, 98–100). This approach has recently been evaluated in patients with SLE within the frame of e.g., the BEAT Lupus (101, 102) and BLISS-BELIEVE (103) trials. The latter, however, failed to show superior disease control with the addition of a single cycle of rituximab on top of belimumab treatment over that achieved by belimumab treatment alone, while more serious infections were seen in the combination group (104). Furthermore, the concept of combining B cell therapies has also been studied in the context of lupus nephritis, e.g., in the Rituximab and Belimumab for Lupus Nephritis (CALIBRATE; NCT02260934) (105) and Synergetic B cell Immunomodulation in SLE (SynBioSe) trials (SynBioSe 1: NCT02284984 (106); SynBioSe 2: NCT03747159). While the CALIBRATE study failed to reach its primary endpoint i.e., achievement of renal response at week 48, biological effects in the form of decreased anti-dsDNA antibody levels and modification of the B cell pool composition favored the addition of belimumab to a therapeutic regimen comprising rituximab and cyclophosphamide. Importantly, no safety signals emerged for this combination.

**Figure 1:**
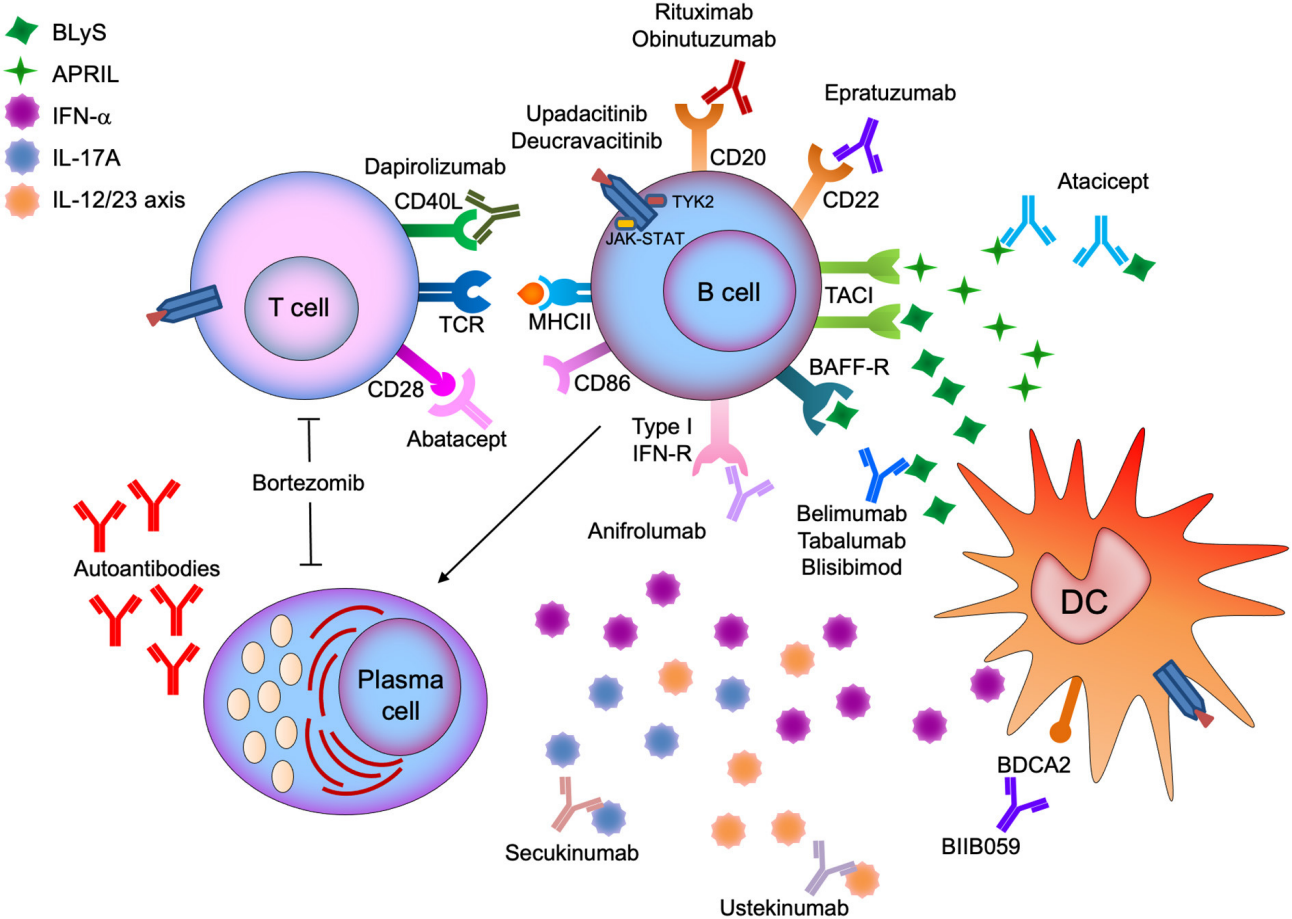
Illustration of the main mode of action of compounds studied for the treatment of systemic lupus erythematosus (SLE) and lupus nephritis (LN) that exert direct or indirect effects on B cells.

A summary of recent or ongoing trials of agents that directly or indirectly alter the B cell constitution in patients with SLE and lupus nephritis is presented in Table 1. Figure 1 illustrates the main mode of action of compounds discussed in the present review.

**Table 1:** Selected drugs studied for SLE and LN that exert direct or indirect effects on B cells.

**Drug name**	**Mechanism**	**Phase**	**Endpoint**	**Results**	**References**
**Adaptive immunity**					
BIIB059	anti-BDCA2	II SLE/CLE	DAS28 w24 CLASI w16	Successful	NCT02847598
		III SLE	SRI-4 w52	Ongoing	NCT04895241 NCT04961567
Obinutuzumab	anti-CD20	II LN	CRR w52	Successful	Furie et al. (46)
		III LN	CRR w76	Ongoing	NCT04221477
Belimumab	anti-BAFF	III LN	PERR w104	Successful	Furie et al. (107)
Rituximab-belimumab	Sequential CD20-BAFF inhibition	II SLE	IgG anti-dsDNA w52	Successful	Shipa et al. (102)
Rituximab-belimumab (CALIBRATE)	Sequential CD20-BAFF inhibition	II LN	Safety (met); Secondary: CR or PR w48 (not met)	Failed	Atisha-Fregoso et al. (105)
Belimumab-rituximab (BLISS-BELIEVE)	Sequential BAFF-CD20 inhibition	III SLE	Remission w52	Failed	Aranow et al. (104)
Dapirolizumab	anti-CD40L	II SLE	BICLA w24	Successful	NCT02804763
		III SLE	BICLA w48	Ongoing	NCT04294667
**Small molecules**					
Baricitinib	anti-JAK1	II SLE	Resolution of arthritis or rash w24	Successful	Wallace et al. (108)
Baricitinib BRAVE I, II	anti-JAK1	III SLE	SRI-4 w52	Terminated	NCT03616912
					NCT03616964
Tofacitinib	anti-JAK1/3	I SLE	Safety	Drug stopped	Hasni et al. (109)
BMS-986165	anti-TYK2	II LN	Safety; Secondary: CRR or PRR w24	Terminated	NCT03943147
Deucravacitinib	anti-TYK2	II DLE, SCLE	Change in CLASI-A w16	Ongoing	NCT04857034
Upadacitinib	anti-JAK1	II SLE	SRI-4 w24	Ongoing	NCT03978520
**Indirect impact on B cells**					
Anifrolumab	anti-IFNAR	III SLE	SRI-4 w52	Failed	Furie et al. (94)
		III SLE	BICLA w52	Successful	Morand et al. (95)
		III LN	CRR w52	Ongoing	NCT05138133
Secukinumab	anti-IL17A	II LN	CRR w52	Ongoing	NCT04181762
Ustekinumab	anti-IL12/23	II SLE	SRI-4 w24	Successful	van Vollenhoven et al. (110)

BAFF, B cell activating factor belonging to the tumor necrosis factor family; BDCA2, blood dendritic cell antigen 2; BICLA, BILAG 2004-Based Composite Lupus Assessment; CLASI, Cutaneous Lupus Erythematosus Disease Area and Severity Index; CLE, cutaneous lupus erythematosus; CR, complete response; CRR, complete renal response; DLE, discoid lupus erythematosus; IFNAR, interferon-α/β receptor; JAK, Janus kinase; LN, lupus nephritis; PERR, Primary efficacy renal response; PR, partial response; PRR, partial renal response; SCLE, subacute cutaneous lupus erythematosus; SLE, systemic lupus erythematosus; SRI, SLE Responder Index; TYK, tyrosine kinase; w, week.

## The prospect of B cells as surveillance tools

Changes in the circulating B cell pool are expected upon B cell targeting therapies and have been described in response to anti-CD20 (22, 39, 111) and anti-BAFF (74, 112, 113) therapies. On the other hand, little data has piled up so far concerning the potential role of B cell changes as measurable biomarkers of response to therapy or as heralders of disease flares.

Recently, we analyzed data from three phase III RCTs of belimumab i.e., BLISS-76 (NCT00410384) (7), BLISS-SC (NCT01484496) (114), and BLISS Northeast Asia (NEA; NCT01345253) (115) and pinpointed specific relative to baseline percentage changes across different circulating CD19^+^ B cell and plasma cell subsets occurring in SLE patients after treatment commencement for active disease with standard therapy with or without add-on belimumab to be associated with treatment response (116) or disease flares (117). Within a pooled cohort of 1712 patients with SLE, treatment responders showed more prominent decreases from baseline through week 52 in CD19^+^CD20^+^CD27^−^ naïve B cells, CD19^+^CD20^−^CD27^bright^ plasmablasts, and CD19^+^CD20^−^CD138^+^ long-lived plasma cells, and a more prominent rapid (through week 8) and early (through week 24) expansion of CD19^+^CD20^+^CD27^+^ memory B cells compared with non-responders (116). Conversely, patients who developed severe flares showed less prominent early decreases in CD19^+^CD20^−^CD138^+^ long-lived plasma cells and CD19^+^CD27^bright^CD38^bright^ SLE-associated plasma cells (117).

In an analysis of B cell changes in relation to renal flare development within the same SLE population from trials of belimumab (unpublished data), patients who developed renal flares showed a more prominent rapid decrease in CD19^+^CD20^+^CD138^+^ short-lived plasma cells and CD19^+^CD20^−^CD27^bright^ plasmablasts compared with patients who did not flare, followed by a subsequent return. Remarkably, more prominent rapid reductions in CD19^+^CD27^−^CD24^bright^CD38^bright^ transitional B cells and CD19^+^CD20^−^CD138^+^ peripheral long-lived plasma cells were associated with a protection against renal flares in belimumab-treated patients.

It is worth noting that B cell changes in these analyses were mainly related to treatment effectiveness rather than the mechanism of the drug employed. However, considering its mode of action, belimumab is expected to impact on B cells of early maturation stages, as shown previously (74, 112, 113, 118), and decreasing B cell counts may be expected to predict favorable responses to belimumab, as previously demonstrated for rituximab in patients achieving adequate B cell depletion (22, 39). Moreover, in a real-life observational study, immunological responses to belimumab preceded overt clinical improvements (74). Thus, since changes in circulating B cell subsets may be expected to occur shortly upon therapy commencement, such changes could prove a useful tool in the treatment and disease monitoring in patients with SLE, complemental to traditional serological biomarkers such as anti-dsDNA and complement levels, which may be less sensitive to change. Lastly, the use of relevant molecules in the prediction of response to therapies is expected to advance treatment selection and monitoring in the future. High BAFF levels, for instance, have been associated with favorable clinical response to treatment with belimumab (72), but also imminent flare in patients with SLE on non-biological standard therapy (119).

## Concluding remarks

SLE is characterized by breach of self-tolerance and constant B cell hyperactivity. While the contribution of B cells to disease initiation is unclear, they have key roles in the pathogenesis of established SLE and the maintenance of the chronic inflammatory milieu. Several B cell targeting therapeutic modalities have an established role in the SLE treatment armamentarium, while other are in the pipeline. Optimized use of B cell altering therapeutic approaches would require effective identification of patients who would be more likely to benefit from B cell therapies based on their clinical and immunological phenotypes. In this regard, the usefulness of B cell and plasma cell levels and alterations as markers of treatment response or flare development has not been thoroughly explored, but recent data show merit and ratify further survey.

## Author contributions

All authors contributed to the manuscript draft, critically reviewed all parts of the manuscript, accepted its final version prior to submission, and account for its content.

## Funding

IP was funded by grants from the Swedish Rheumatism Association (R-941095), King Gustaf V's 80-year Foundation (FAI-2020-0741), Professor Nanna Svartz Foundation (2020-00368), Ulla and Roland Gustafsson Foundation (2021-26), Region Stockholm (FoUI-955483) and Karolinska Institutet. CS was funded by the Swedish Rheumatism Association (R-939149), Region Östergötland (ALF grants; RÖ-960604), King Gustaf V's 80-year Anniversary foundation (FAI-2020-0663), and King Gustaf V and Queen Victoria's Freemasons foundation. The funders had no role in the design of the study, the analyses or interpretation of data, or the writing of the manuscript.

## Conflict of interest

Author IP has received research funding and/or honoraria from Amgen, AstraZeneca, Aurinia Pharmaceuticals, Elli Lilly and Company, Gilead Sciences, GlaxoSmithKline, Janssen Pharmaceuticals, Novartis, and F. Hoffmann-La Roche AG. The remaining authors declare that the research was conducted in the absence of any commercial or financial relationships that could be construed as a potential conflict of interest.

## Publisher's note

All claims expressed in this article are solely those of the authors and do not necessarily represent those of their affiliated organizations, or those of the publisher, the editors and the reviewers. Any product that may be evaluated in this article, or claim that may be made by its manufacturer, is not guaranteed or endorsed by the publisher.
